# Speech Depression Screening via Multi-Scale Feature Enhancement and Emotion-Aware Contrastive Learning

**DOI:** 10.3390/bioengineering13070739

**Published:** 2026-06-25

**Authors:** Zhengyuan Chen, Meihong Wu

**Affiliations:** 1 School of Informatics, Xiamen University, Xiamen 361102, China; 37220222203569@stu.xmu.edu.cn; 2 Hearing and Speech Laboratory, Xiamen University, Xiamen 361005, China

**Keywords:** depression recognition, subclinical depression, speech analysis, Wav2Vec 2.0, multi-scale convolution, contrastive learning

## Abstract

Speech-based depression screening is a promising non-invasive approach, but short spontaneous-speech segments often contain weak acoustic cues, fragmented semantic context, and overlapping representations between depressed and non-depressed participants. This study addresses this problem by proposing a preliminary short-segment acoustic screening framework that integrates multi-scale feature enhancement and reference-enhanced contrastive calibration. Based on the self-supervised pretrained Wav2Vec 2.0 model, a Multi-Scale Convolution (MSC) module captures short-term vocal fluctuations and longer-range prosodic patterns. A Reference-Enhanced Contrastive Learning (ReCLR) mechanism further calibrates latent acoustic representations using external emotion-reference features. Whisper-based transcription features are also incorporated to test whether short-segment semantic information can complement acoustic cues. In the participant-independent DAIC-WOZ evaluation, the proposed framework achieved an accuracy of 0.7021, a specificity of 0.8485 for non-depressed participants, and a sensitivity of 0.3571 for depressed participants. These results indicate that multi-scale acoustic enhancement and emotion-reference contrastive calibration may improve specificity-oriented short-segment screening, but the limited depressed-class sensitivity shows that the framework remains preliminary and requires stronger threshold calibration, training stability, and external validation before practical deployment.

## 1. Introduction

Major Depressive Disorder (MDD) is one of the most common mental disorders worldwide and remains a major public health challenge because of its disability burden and suicide risk [1,2]. Current clinical assessment relies mainly on subjective rating scales, such as PHQ-9 and HAMD, together with semi-structured interviews by mental health professionals. These procedures are clinically indispensable, but they can be affected by self-report bias, interview context, and limited access to trained clinicians. This gap between population-level screening needs and available clinical resources motivates objective, non-invasive, and scalable digital biomarkers for preliminary mental health screening.

Speech is a promising digital biomarker because it carries emotional, cognitive, and neurophysiological information. Depression-related psychomotor retardation can be reflected in reduced fundamental-frequency dynamics, slower speech rate, prolonged pauses, and high-frequency energy attenuation [3,4]. Early speech-based depression studies relied mainly on hand-crafted low-level descriptors (LLDs) [5], whereas recent deep learning models have improved representation learning with CNNs, LSTMs, and self-supervised learning (SSL) frameworks [6,7]. In particular, Wav2Vec 2.0 can learn contextual acoustic representations from large-scale unlabeled speech and has shown promise for low-resource mental health speech tasks [6,8,9].

The specific problem addressed in this study is short-segment spontaneous-speech screening. In this setting, a model must infer participant-level depression risk from brief speech windows that may contain only weak pathological cues. This problem is difficult for three reasons. First, acoustic abnormalities in mild or subclinical conditions can be subtle and easily smoothed by global utterance-level aggregation [10,11]. Second, depressive speech is cross-scale: local vocal changes may occur over milliseconds, whereas pausing, prosodic slowing, and discourse stagnation unfold over longer intervals [12]. Third, depressed and non-depressed speech representations can overlap substantially in the latent space, causing conservative decision boundaries and missed positive cases.

Recent studies have addressed related parts of this problem through self-supervised acoustic representation learning, hierarchical temporal modeling, speaker disentanglement, and multimodal fusion. Wav2Vec-based studies and localized self-supervised models have explored robust acoustic features under limited clinical data [9,10]. Voice–text fusion [13], multimodal sentiment modeling [14], speaker-leakage control [15,16], and audio–video–text fusion [17] have also been investigated. Multi-scale and hierarchical approaches, including STFN [18] and dynamic-window attention-based Transformer models [19], further emphasize temporal structure. Multimodal frameworks such as SIMMA [20] have explored crossmodal alignment, while language-model-based methods have incorporated psychological knowledge into spoken-language analysis [21]. However, these studies differ in modality, segmentation strategy, dataset split, and evaluation protocol. For 10 s spontaneous-speech screening, it remains unclear whether short-window acoustic enhancement and emotion-reference calibration can improve participant-level screening, and whether short text segments provide stable semantic cues.

**Figure 1:**
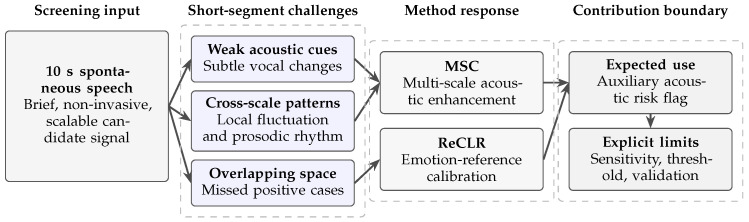
Motivation and problem–method mapping of the proposed short-segment speech depression screening framework. The figure links the background screening input to the main short-segment challenges, the corresponding MSC-ReCLR design responses, and the expected contribution with its practical boundary.

Figure 1 summarizes the short-segment screening problem, the method response, and the intended contribution boundary.

**Figure 2:**
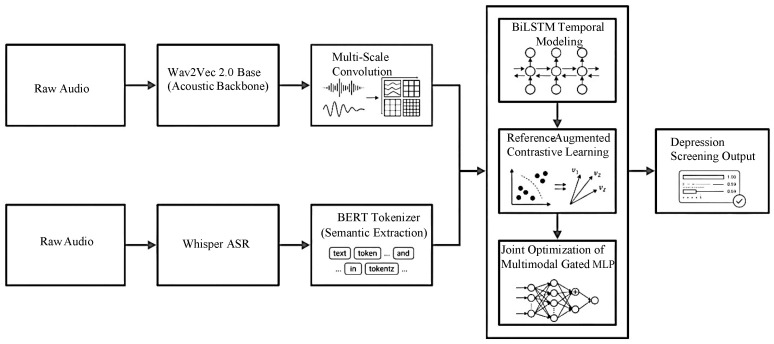
Schematic illustration of the multi-scale fusion and contrastive enhancement framework.

To address this gap, this study proposes a preliminary depression acoustic screening framework integrating Multi-Scale Convolution and Reference-enhanced Contrastive Learning (MSC-ReCLR). Wav2Vec 2.0 is used as the acoustic backbone. The MSC module captures acoustic patterns ranging from local phoneme-level variation to longer prosodic rhythm through receptive fields of different sizes. The ReCLR mechanism [22] calibrates latent acoustic representations using external emotion-reference features extracted from a pretrained speech emotion model [23,24]. In addition, Whisper-based transcription features are included to test whether short-segment semantic information can complement acoustic cues [21]. The main participant-independent evaluation is conducted on DAIC-WOZ, while MODMA is reported as supplementary evidence because of its different language and recording setting. Figure 2 gives an overview of the proposed multi-scale fusion and contrastive enhancement framework.

In summary, the main contributions of this paper are as follows:

- We propose MSC-ReCLR, a short-segment acoustic screening framework that combines Wav2Vec 2.0, multi-scale temporal enhancement, and emotion-reference contrastive calibration.
- We evaluate the framework under participant-independent DAIC-WOZ testing and report patient-level accuracy, sensitivity, specificity, AUC-ROC, confusion matrices, bootstrap confidence intervals, and threshold sensitivity.
- We analyze the practical limitations of short-segment screening, including depressed-class false negatives, feature-space overlap, repeated-seed instability, and the limited utility of simple Whisper-based semantic fusion.
- We clarify the intended application boundary of the framework as a preliminary acoustic screening aid rather than a stand-alone clinical diagnostic system.

## 2. Materials and Methods

### 2.1. Datasets

This study uses two public speech-based depression datasets: DAIC-WOZ (English) and MODMA (Chinese). DAIC-WOZ [25] provides structured clinical interview recordings and depression severity annotations based on the PHQ-8 scale. MODMA contains speech samples from clinically diagnosed depressed patients and healthy control participants. In the present revision, DAIC-WOZ remains the primary benchmark for participant-independent evaluation, whereas MODMA is reported as a supplementary dataset for assessing whether the same short-segment preprocessing and patient-level reporting protocol can be applied to a dataset with a different language, task structure, and recording protocol.

Considering that real clinical interview recordings are inevitably mixed with the interviewer’s guiding words and environmental noise, this study designed a strict preprocessing pipeline for DAIC-WOZ. First, a deep neural network-based Pyannote Speaker Diarization toolkit [26] was used for unsupervised speaker separation to remove the interviewer’s speech and retain participant speech. Subsequently, participant speech was segmented into fixed 10 s windows to capture local temporal acoustic cues and to reduce the effect of highly variable recording duration. All input waveforms were uniformly resampled to 16 kHz and normalized before being fed into the network. For MODMA, the processed metadata contain the same participant-level labels, 10 s segment labels, and train/development/test split identifiers. Because the currently available MODMA-processed files do not include diarization outputs, MODMA is described separately and interpreted cautiously unless the recording protocol confirms that the speech samples are single-speaker or already speaker-clean.

For the DAIC-WOZ experiments, all splits were performed at the participant level before segment-level processing. Therefore, all 10 s segments generated from the same participant remained in the same training, development, or testing split. As shown in Table 1, no participant ID appeared in more than one split, which avoided participant-level data leakage between training and testing. Table 2 further summarizes the symptom severity distribution in each split.

For the processed MODMA metadata, the participant-level split also avoids overlap across training, development, and testing sets. The processed MODMA subset contains 52 participants and 1860 ten-second segments in total, as summarized in Table 3 and Table 4. Given the differences between MODMA and DAIC-WOZ in language, elicitation tasks, and recording conditions, the MODMA results are treated as supplementary evidence rather than as definitive external clinical validation.

**Figure 3:**
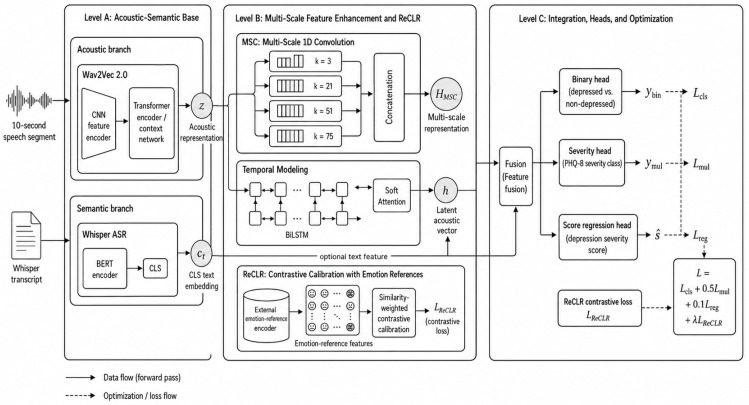
Dual-tower cascaded framework for speech-based depression screening and multi-task joint optimization. The acoustic branch extracts Wav2Vec 2.0 representations and applies MSC, BiLSTM, and soft attention to obtain the latent acoustic vector, while the optional semantic branch extracts Whisper–BERT text embeddings for feature-level fusion. ReCLR calibrates the acoustic representation using external emotion-reference features, and the model is jointly optimized with binary classification, PHQ-8 severity classification, score regression, and contrastive calibration losses.

In terms of the text modality, this study abandoned the traditional transcription method that highly relies on manual proofreading, and instead adopted the robust large-scale pretrained automatic speech recognition (ASR) model Whisper to directly extract transcription text from the cleaned patient speech segments [27]. The transcribed sequence was then mapped into discrete tokens by the BERT Tokenizer, and uniformly truncated or zero-padded to a fixed sequence length $L_{text}$, achieving efficient tensorized batch training. The proposed dual-tower cascaded model and its multi-task joint optimization framework are shown in Figure 3.

### 2.2. Speech Representation Based on Self-Supervised Priors and Multi-Scale Feature Extraction

The acoustic representation of depression is highly heterogeneous. To extract robust underlying acoustic cues, this study selected the Wav2Vec 2.0 Base model as the main acoustic feature extractor (backbone). Compared with traditional hand-crafted LLDs (such as MFCCs or eGeMAPS), Wav2Vec 2.0 can implicitly capture rich context-aware and prosodic features directly from the raw one-dimensional waveform through the self-supervised pretraining mechanism of a masked language model (MLM) [6,28,29].

**Figure 4:**
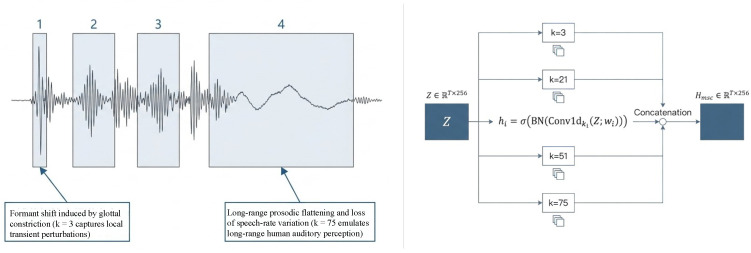
Schematic of MSC addressing temporal asymmetry in depressive speech.

Aiming at the weak pathological fluctuations that are often masked by global averaging in subclinical or mild depression speech [30], this framework incorporates a Multi-Scale Convolution (MSC) module in parallel on top of the Transformer encoder of Wav2Vec 2.0. This module consists of multiple one-dimensional convolution (1D-CNN) branches with step-wise increasing receptive fields, designed to simultaneously capture cross-scale features ranging from microscopic phoneme pronunciation anomalies (short window) to macroscopic utterance pauses (long window), as shown in Figure 4.

Let $X\in R^{T\times d}$ denote the sequence features output by Wav2Vec 2.0. The multi-scale fusion process can be formalized as:

(1) $$F_{msc}=\mathrm{Concat}\left[{\mathrm{Conv}}_{k_{1}}\left(X\right),{\mathrm{Conv}}_{k_{2}}\left(X\right),\ldots ,{\mathrm{Conv}}_{k_{n}}\left(X\right)\right]$$

where ${\mathrm{Conv}}_{k_{i}}\left(\cdot \right)$ denotes a convolution operation with kernel size $k_{i}$ and stride 1 (equipped with Batch Normalization and GELU activation function), and *n* is the total number of scale branches. The fused feature tensor $F_{msc}$ is then fed into a pooling layer to reduce the spatial dimensionality. Compared with a single receptive field structure, the MSC module greatly improves the spatial resolution of the network for high-frequency weak pathological information.

### 2.3. Temporal Modeling and Reference-Enhanced Contrastive Calibration

**Figure 5:**
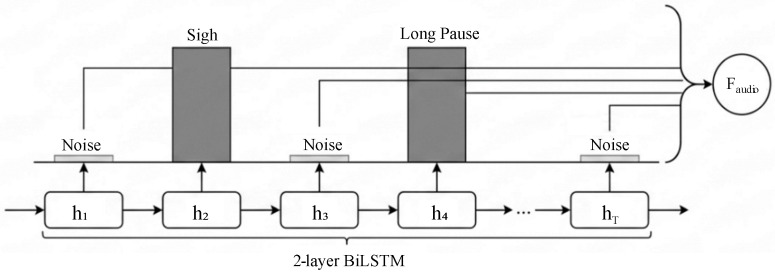
Schematic illustration of temporal modeling with an adaptive soft attention mechanism.

After extracting frame-level multi-scale features, this study utilizes a two-layer Bidirectional Long Short-Term Memory network (BiLSTM) [31] to capture the long-range temporal dependencies among the feature sequence. To enable the model to dynamically focus on the most informative speech segments (such as sighs, long pauses, or sudden changes in speech rate) while effectively suppressing meaningless noise frames in spontaneous speech, we integrated a soft attention mechanism [32] after the output layer of the BiLSTM, as shown in Figure 5.

Let $h_{t}$ denote the hidden state at time step *t*. The attention weight $\alpha_{t}$ is calculated as follows:

(2) $$e_{t}=v^{⊤}\tanh\left(Wh_{t}+b\right)$$

(3) $$\alpha_{t}=\frac{\exp(e_{t})}{\sum_{j=1}^{T}\exp\left(e_{j}\right)}$$

where W and v are learnable projection matrices and context vectors, and the final acoustic-level representation is calculated as $F_{audio}=\sum_{t=1}^{T}\alpha_{t}h_{t}$.

To reduce feature-space overlap between depressed and non-depressed speech representations, we introduced a Reference-enhanced Contrastive Learning (ReCLR) mechanism. In the implementation, the acoustic representation $z_{i}$ is obtained from the MSC–BiLSTM–attention encoder, while an external emotion-reference representation $r_{i}$ is extracted from a pretrained emotion representation model (‘superb/wav2vec2-base-superb-er’). After normalization, the acoustic similarity and emotion-reference similarity for a mini-batch are computed as:

(4) $$s_{ij}=\frac{z_{i}^{⊤}z_{j}}{\tau },\;e_{ij}=r_{i}^{⊤}r_{j}$$

where τ is the temperature parameter. For each anchor sample, samples with the same binary label are treated as positive pairs, while samples from the opposite class are treated as negative pairs. The emotion-reference similarity is used to weight the penalty imposed on negative pairs:

(5) $$L_{ReCLR}^{\left(i\right)}=-\log\frac{\sum_{j}I\left(y_{j}=y_{i}\right)\exp\left(s_{ij}\right)}{\sum_{j}I\left(y_{j}=y_{i}\right)\exp\left(s_{ij}\right)+\sum_{j}I\left(y_{j}\neq y_{i}\right)\exp\left(s_{ij}\right)\exp\left(e_{ij}\right)+\epsilon }.$$

This formulation does not use manually specified class prototypes; instead, it uses an external emotion-reference encoder to modulate the contrastive penalty within each mini-batch. The entire framework adopts a joint optimization strategy, and the total loss function is defined as:

(6) $$L_{total}=L_{cls}+0.5L_{multi}+0.1L_{reg}+\lambda L_{ReCLR}$$

where $L_{cls}$ is the binary cross-entropy classification loss, $L_{multi}$ is the multi-class severity classification loss, $L_{reg}$ is the PHQ-8 score regression loss, and λ=0.15 is used to balance the contrastive calibration term.

### 2.4. Acoustic–Semantic Multimodal Fusion and Experimental Evaluation Settings

The semantic content and acoustic prosody of language possess natural complementarity in expressing mental states. To this end, this study constructed a Feature-level multimodal synergistic fusion module. The global semantic embedding (CLS Token) extracted by the pretrained BERT model [33] is represented as $F_{text}$. Since the acoustic and semantic features reside in different high-dimensional manifold spaces, we employ gated nonlinear projections to align and concatenate them [34]:

(7) $$F_{fused}=\phi \left(\left[F_{audio};F_{text}\right]\right)$$

where [·;·] denotes the concatenation operation on the feature dimension, and ϕ(·) is a Multi-Layer Perceptron (MLP) mapping function including Dropout regularization. $F_{fused}$ is finally fed into a Softmax classification head to output the probability of depression.

In the experimental implementation, all models were built using the PyTorch 2.0 framework and trained on a single NVIDIA RTX 3090 GPU with 24 GB of VRAM. The training process adopted the AdamW optimizer to alleviate the risk of overfitting caused by weight decay, with the initial learning rate set to $2\times 10^{-5}$ and coupled with a cosine annealing learning rate scheduling strategy. To ensure participant-independent evaluation, the official training, development, and testing splits were preserved at the participant level. For patient-level evaluation, the predicted depression probabilities of all 10 s segments from the same participant were averaged, and a threshold of 0.5 was used to obtain the final binary patient-level prediction. In addition to accuracy and F1 score, we report depressed-class precision, depressed-class recall (sensitivity), non-depressed recall (specificity), AUC-ROC, average precision, and the patient-level confusion matrix.

## 3. Results

### 3.1. Main Experimental Results

To evaluate the proposed MSC-ReCLR framework, we first compared the internal model variants under the same participant-independent DAIC-WOZ protocol. We then compared the framework with traditional machine learning baselines and positioned it relative to recent speech-based depression detection studies. Because published studies differ in modality, segmentation strategy, dataset split, and evaluation protocol, the recent-study comparison is used for methodological positioning rather than as a strict performance leaderboard.

Table 5 shows that the baseline Wav2Vec model achieved an accuracy of 0.5532 and a non-depressed recall of 0.55. After introducing MSC, the accuracy increased to 0.6170 and non-depressed recall increased to 0.67. After adding ReCLR, the accuracy reached 0.7021 and non-depressed recall increased to 0.85. Non-depressed recall corresponded to specificity in the binary screening setting, not to sensitivity for depressed participants. The main observed improvement was therefore better identification of non-depressed participants.

Table 6 shows that some traditional baselines achieved competitive accuracy under the same experimental setting. For example, Max + Logistic Regression reached an accuracy of 0.743. However, the corresponding F1 scores remained limited across these baselines. Together with Table 7, these results indicate that MSC-ReCLR should not be interpreted as a universal state-of-the-art model. Its value is more specific: it tests whether multi-scale acoustic enhancement and emotion-reference calibration can improve short-segment participant-level screening under a controlled protocol.

As shown in Table 8 and Table 9, the final model correctly classified 28 of 33 non-depressed participants, but it detected only 5 of 14 depressed participants. The depressed-class sensitivity was 0.3571 and the AUC-ROC was 0.5411. The improvement should therefore be interpreted as specificity-oriented acoustic screening rather than robust depressed-case detection. This distinction is important for practical use because an early screening tool should usually prioritize sensitivity before it can support real-world triage.

### 3.2. Statistical Uncertainty and Threshold Sensitivity

To further assess the uncertainty of the patient-level results, we performed a non-parametric bootstrap analysis at the participant level. Specifically, the 47 test participants were resampled with replacement, and the main evaluation metrics were recalculated for each bootstrap sample. Table 10 reports the point estimates and 95% bootstrap confidence intervals of the final MSC-ReCLR model.

The wide confidence intervals reflect the small number of depressed participants in the test set and indicate that the reported patient-level metrics should be interpreted cautiously. In particular, the confidence interval of depressed-class sensitivity remains broad, confirming that the model’s ability to detect positive cases is not yet statistically stable.

Because the model showed a clear sensitivity–specificity trade-off, we also evaluated patient-level predictions under different decision thresholds while keeping the predicted probabilities fixed. As shown in Table 11, lowering the threshold increased depressed-class sensitivity, but reduced specificity and overall accuracy. The fixed threshold of 0.5 therefore represents a conservative operating point with relatively high specificity but limited sensitivity.

In addition to the bootstrap and threshold analyses, we performed supplementary repeated-seed diagnostic runs for the ReCLR model under the same fixed threshold of 0.5. These runs were not used to claim performance robustness because all three seeds collapsed to all-negative patient-level predictions, with accuracy of 0.7021, sensitivity of 0.0000, specificity of 1.0000, depressed-class F1 of 0.0000, and a predicted-positive rate of 0.0000. The patient-level probability distributions were also concentrated below the threshold, with mean probabilities of approximately 0.404, 0.362, and 0.365 across the three seeds. This diagnostic result indicates that the current model is sensitive to random initialization and fixed-threshold calibration; therefore, repeated-seed behavior is treated as a limitation rather than as positive evidence of stable performance.

It is worth noting that the multimodal model fused with Whisper transcription text did not provide a reliable improvement. Its non-depressed recall was only 0.0606, showing a tendency to over-predict the depressed class. A possible reason is that, under 10 s segmentation, Whisper transcriptions often contain fragmented words and lack complete semantic context, making it difficult for BERT to extract stable semantic representations. This result suggests that simple feature-level multimodal fusion may be unreliable when only short spontaneous speech segments are available.

On the supplementary MODMA test split, the model achieved an accuracy of 0.7273 and an F1 score of 0.7273. This result indicates that the proposed short-segment acoustic pipeline can be applied to the processed MODMA subset. However, it should be interpreted cautiously because MODMA differs from DAIC-WOZ in language, recording protocol, label definition, and task structure. Therefore, this result is reported as supplementary dataset evidence, while stronger claims of external or cross-lingual validation require a predefined cross-dataset protocol and additional full-metric reporting.

### 3.3. Positioning Against Recent Studies

The comparison in Table 7 clarifies how the present framework relates to recent work. Existing studies have shown the usefulness of pretrained speech representations [9,10], hierarchical temporal modeling [18,19], and multimodal alignment [20]. The present study differs by focusing on participant-independent 10 s acoustic screening and by explicitly reporting patient-level uncertainty, threshold sensitivity, and error behavior. This setting is clinically relevant for scalable screening, but it is also more restrictive than long-context or multimodal interview analysis. The present results therefore support a narrower conclusion: MSC-ReCLR provides a preliminary framework for specificity-oriented short-segment acoustic screening, while stronger depressed-case detection still requires improved calibration, larger external cohorts, and more stable patient-level aggregation.

### 3.4. Sensitivity Analysis Across Depression Severity Levels

To further explore the model’s ability to recognize interviewees with different levels of depression, this study conducted a grouped analysis of the test samples based on PHQ-8 scores, with the experimental results shown in Table 12.

The grouped results should be interpreted together with the binary labeling protocol of DAIC-WOZ. Participants with PHQ-8 scores of 5–9 are shown as a subclinical group for descriptive analysis, but they are still assigned to the binary non-depressed class under the PHQ-8 binary threshold used in the dataset. Therefore, the accuracy of 1.0000 in this subgroup indicates that the model classified all 11 mildly symptomatic but binary-negative participants consistently with their binary labels; it should not be interpreted as validated positive detection of subclinical depression. In contrast, the moderate-to-severe group contains binary-positive depressed participants, and the final model achieves only 0.3571 accuracy in this group. This result is consistent with the low depressed-class sensitivity reported in Table 8 and indicates that the current model still fails to identify a substantial proportion of depressed participants.

### 3.5. Post Hoc Interpretability and Error Analysis

To further examine the behavior of the final model, we conducted a post hoc interpretability and error analysis using the main checkpoint that reproduced the patient-level results reported in Table 8. Patient-level probabilities, patient-level embeddings, and segment-level prediction traces were extracted from the DAIC-WOZ test set. This analysis was used to characterize model behavior and failure modes, rather than to claim clinical explainability.

**Figure 6:**
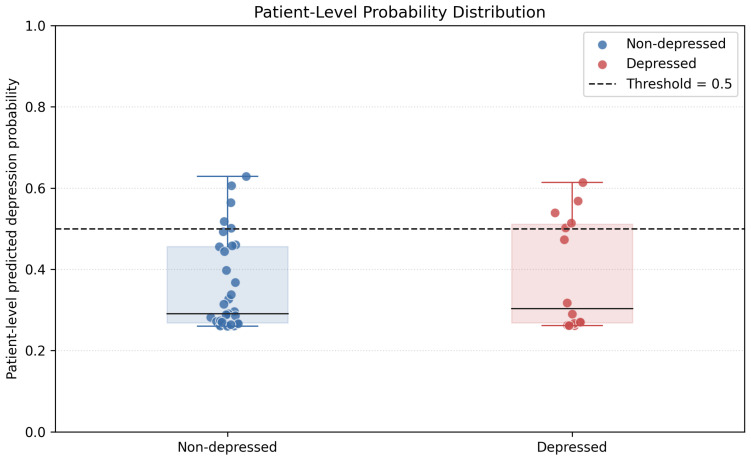
Patient-level predicted probability distribution of the final MSC-ReCLR model on the DAIC-WOZ test set. The dashed line indicates the fixed decision threshold of 0.5.

The patient-level probability distribution shown in Figure 6 indicates that the predicted probabilities of depressed and non-depressed participants showed substantial overlap. Many depressed participants received probabilities below the fixed threshold, while several non-depressed participants were assigned probabilities slightly above the threshold. This pattern is consistent with the limited AUC-ROC and the low depressed-class sensitivity reported above, and it explains why threshold adjustment changes the sensitivity–specificity balance without fully resolving the class-separation problem.

**Figure 7:**
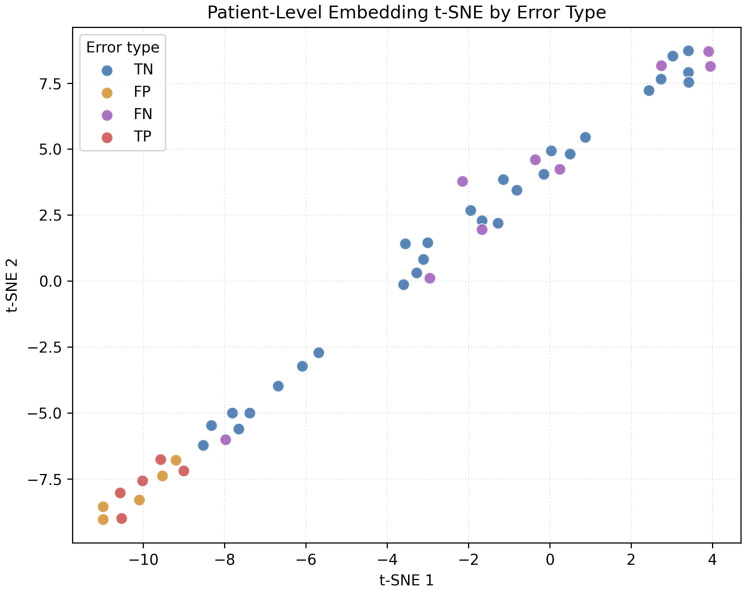
t-SNE visualization of patient-level embeddings colored by prediction outcome. TN, FP, FN, and TP denote true negative, false positive, false negative, and true positive, respectively.

The t-SNE visualization in Figure 7 further shows that the learned patient-level embeddings did not form a clearly separated depressed/non-depressed structure on the test set. False-negative and false-positive cases were located near correctly classified samples rather than appearing as isolated outliers, indicating that the representation space remained partially entangled across diagnostic groups. Error-case inspection also showed that the nine false-negative cases all belonged to the moderate-to-severe PHQ-8 group, including several participants with high PHQ-8 scores. Therefore, the model’s missed detections cannot be attributed only to mild or borderline symptom severity. Segment-level probability traces showed that several errors reflected stable patient-level score tendencies across many speech segments, rather than being caused by a single anomalous segment. Overall, the interpretability analysis supports the conclusion that the current model is conservative at the fixed threshold and still lacks a sufficiently discriminative representation for reliable depressed-case detection.

We further merged the patient-level test predictions with the available DAIC-WOZ metadata for a descriptive error analysis. The split label files provided PHQ-8 scores and binary gender codes, but did not include age information. Stratifying the 47 test participants by the available gender code yielded similar overall accuracy for the two groups (0.7083 for gender = 0 and 0.6957 for gender = 1), while depressed-class sensitivity was 0.4286 and 0.2857, respectively. Because each subgroup contained only a small number of depressed participants, these values are reported only as descriptive error analysis results rather than as a formal fairness or subgroup validation test. The PHQ-8 score distribution by error type was more informative: false-negative participants had a mean PHQ-8 score of 16.67, whereas true-positive participants had a mean score of 13.40. This pattern again indicates that several missed detections occurred among participants with clinically meaningful symptom severity, reinforcing the need for improved depressed-case sensitivity and better calibrated patient-level decision rules.

## 4. Discussion

### 4.1. Interpretation of Main Findings

This study addresses a specific problem in speech-based depression screening: participant-level risk inference from short spontaneous-speech segments. Under a participant-independent DAIC-WOZ evaluation protocol, MSC-ReCLR improved overall accuracy and specificity compared with the Wav2Vec 2.0 baseline. The main gain was not a balanced improvement across both classes. Instead, the framework became more conservative and more accurate for non-depressed participants, while depressed-class sensitivity remained limited.

This finding gives the present work a clear but narrow interpretation. MSC-ReCLR provides preliminary evidence that multi-scale acoustic encoding and emotion-reference contrastive calibration can improve specificity-oriented short-segment screening. It does not yet provide sufficient evidence for reliable positive-case detection. This distinction is central because missed depressed participants are more harmful than false positives in many screening workflows.

### 4.2. Why the Proposed Method Helps and Where It Fails

The MSC module is motivated by the cross-scale temporal hierarchy of depressive speech. Unlike a single fixed receptive field, the parallel multi-scale design can encode short-window acoustic variations that may correspond to local spectral fluctuation or articulatory instability, while larger kernels provide access to longer prosodic rhythm and pausing patterns. In this sense, MSC provides a simple mechanism for combining local and longer-range acoustic evidence before BiLSTM-attention temporal aggregation.

The ReCLR mechanism further calibrates the latent acoustic space using external emotion-reference features. In the current implementation, these reference features are extracted from a pretrained speech emotion representation model rather than from manually defined clinical anchors. The role of ReCLR is therefore best understood as emotion-reference-guided contrastive calibration: it encourages the acoustic representation space to reflect both diagnostic labels and emotion-related similarity structure. This may help reduce overlap between depressed and non-depressed segment representations, but the patient-level confusion matrix shows that 9 of 14 depressed participants were still missed. Thus, feature-space calibration remains incomplete and requires further optimization.

The post hoc embedding, probability, and metadata-based error analyses provide additional evidence for this incomplete calibration. Although the final model achieved relatively high specificity, patient-level probabilities from the two binary classes overlapped substantially, and the t-SNE visualization did not show a clean separation of true-positive, false-negative, false-positive, and true-negative cases. Several false-negative participants had moderate-to-severe PHQ-8 scores, suggesting that missed detections were not limited to mild or ambiguous cases. The available gender-coded subgroup analysis was descriptive only and did not provide a sufficient sample size for a formal fairness assessment. These findings indicate that MSC-ReCLR can reshape the acoustic representation space to some extent, but the present training objective and aggregation strategy do not yet produce a reliably separable patient-level manifold.

### 4.3. Comparison with Recent Studies

Recent speech-based depression detection studies have explored several complementary directions. Pretrained speech models can improve representation learning under limited data [9,10]. Hierarchical and dynamic-window temporal models emphasize that depression-related cues are distributed across multiple time scales [18,19]. Multimodal systems can integrate acoustic, visual, and textual information when a longer and better-aligned interview context is available [20]. The present work is consistent with these directions, but it focuses on a narrower short-segment acoustic setting.

The comparison with recent studies should therefore be interpreted methodologically rather than competitively. The current framework is different from long-context multimodal systems because it deliberately examines 10 s spontaneous-speech segments and patient-level aggregation. This setting makes the model easier to apply to short screening recordings, but it also removes discourse-level context that may be important for depression detection. The results show that MSC-ReCLR can improve specificity compared with the internal Wav2Vec baseline, but they also show that short-segment acoustic modeling alone is not sufficient for reliable depressed-case detection.

### 4.4. Semantic Truncation and Multimodal Conflict

The multimodal fusion experiment revealed a practical limitation of fixed 10 s segmentation. Directly fusing Whisper-based text and acoustic features did not improve final screening performance. A likely explanation is that isolated short segments often lack complete semantic context. In clinical interviews, depressive information may emerge through long-range discourse patterns, hesitation, topic development, and cognitive fatigue rather than through isolated words. When the dialog is divided into short windows, the text branch may encode fragmented lexical cues rather than stable discourse-level depressive markers.

This semantic truncation effect also provides a cautious explanation for the uneven severity-group results. Participants with PHQ-8 scores of 5–9 are binary non-depressed in the DAIC-WOZ protocol, and their high subgroup accuracy should not be interpreted as validated detection of subclinical depression. In contrast, moderate-to-severe depressed participants may exhibit longer pauses, global slowing, and long-range discourse disruptions that are not fully preserved in a 10 s window. The observed 0.3571 accuracy in the moderate-to-severe group is therefore consistent with the low depressed-class sensitivity and indicates that local acoustic modeling alone is insufficient for robust detection of all depressed cases.

### 4.5. Practical Application and Deployment Requirements

The practical value of this work lies in its potential role as an auxiliary screening component, not as a replacement for clinical assessment. A short-segment acoustic screening model could be used in remote mental health monitoring, preliminary risk triage, or interview-assist systems where speech is already collected with consent. In industry-oriented settings, the framework could be integrated into telemedicine platforms, digital mental health services, or occupational wellness programs as a low-burden acoustic risk flag. Its advantages are non-invasiveness, low acquisition cost, and compatibility with repeated monitoring. In such workflows, the model would provide a risk signal that prompts further assessment rather than a diagnostic decision.

Several conditions must be satisfied before such use is realistic. First, depressed-class sensitivity must be improved because missed positive cases are unacceptable for stand-alone screening. Second, decision thresholds must be calibrated prospectively for the target population and clinical workflow. Third, the model must be validated on larger external and multi-center cohorts with different languages, recording devices, and interview protocols. Fourth, privacy protection, informed consent, secure data storage, and human review must be built into any deployment pipeline. Finally, clinical use would require integration with existing instruments such as PHQ-9, HAMD, and professional interviews.

### 4.6. Sensitivity–Specificity Trade-Off and Limitations

The relatively low depressed-class F1 score should be interpreted in relation to the sensitivity–specificity trade-off. In the present evaluation, the fixed patient-level threshold of 0.5 led to high specificity but limited sensitivity, meaning that the model was conservative in assigning depressed labels. For an early screening setting, higher sensitivity is usually desirable because missed depressed cases may delay further assessment, whereas false positives can be filtered through subsequent clinical interviews. However, the threshold analysis showed that increasing sensitivity by lowering the decision threshold also increased false positives and reduced specificity. Therefore, the current results do not indicate a clinically optimized operating point; rather, they highlight the need for systematic threshold calibration, cost-sensitive learning, and external validation before practical screening deployment.

The bootstrap confidence intervals further show that the patient-level estimates remain uncertain because of the limited number of test participants, especially the 14 depressed participants in the DAIC-WOZ test set. In addition, supplementary repeated-seed diagnostic runs of the ReCLR model under the fixed 0.5 threshold collapsed to all-negative patient-level predictions across three random seeds, with zero predicted positive cases. The corresponding mean patient-level probabilities were concentrated near the decision threshold but remained below it, indicating sensitivity to random initialization and fixed-threshold calibration. This diagnostic finding was not used as evidence of performance improvement; instead, it reinforced the need for more stable training objectives, calibrated decision thresholds, and patient-level model selection criteria. Taken together, the statistical analyses suggest that the proposed framework captures some acoustic information useful for specificity-oriented screening, but its positive-case detection is not yet robust enough for clinical use.

Several limitations should be acknowledged. First, although the final model reached an accuracy of 0.7021 and a specificity of 0.8485, the depressed-class sensitivity was only 0.3571 and the AUC-ROC was 0.5411, indicating limited positive-case detection and ranking ability. Second, the dataset remains relatively small and imbalanced; although patient-level bootstrap confidence intervals and threshold sensitivity analyses are reported, stronger statistical validation still requires larger external cohorts and prospectively defined operating thresholds. Third, the repeated-seed diagnostics indicate that the ReCLR training and fixed-threshold decision rule are not yet sufficiently stable, so repeated-seed results should be interpreted as a limitation rather than as evidence of robust performance. Fourth, the post hoc interpretability analysis was limited to probability distributions, embedding visualization, metadata-linked descriptive error analysis, and error-case inspection; it helped explain current model behavior but did not establish clinically actionable explainability or formal demographic fairness. Age information was not available in the inspected DAIC-WOZ split labels, and the gender-coded subgroup analysis was underpowered. Fifth, although MODMA is included as a supplementary dataset, differences in language, task design, recording conditions, and label definition limit direct comparability with DAIC-WOZ. Sixth, the 10 s segmentation strategy improves local acoustic modeling but may discard long-range pauses, cognitive fatigue, and discourse-level information that are clinically relevant for moderate-to-severe depression. Future work should focus on improving depressed-class sensitivity through threshold calibration on the development set, class-balanced or focal objectives, repeated-seed training stability checks, and more stable patient-level aggregation strategies. In addition, long-context acoustic–semantic modeling, stronger interpretability methods, lightweight deployment strategies, and multi-center external validation are necessary before this framework can be considered for real-world clinical screening.

## 5. Conclusions

This study proposed MSC-ReCLR, a spontaneous-speech acoustic screening framework designed for short-segment depression risk analysis. The framework combines Wav2Vec 2.0 acoustic representations, Multi-Scale Convolutional encoding, BiLSTM-based temporal modeling, and emotion-reference contrastive calibration. Its central purpose is to examine whether cross-scale acoustic enhancement and latent-space calibration can improve participant-level screening under 10 s spontaneous-speech conditions.

Participant-independent evaluation on DAIC-WOZ showed that MSC-ReCLR improved overall accuracy and specificity compared with the baseline model. However, the depressed-class sensitivity remained limited, and the PHQ-8 5–9 subgroup result should be interpreted only within the binary labeling protocol of DAIC-WOZ. Post hoc interpretability and metadata-linked error analyses further showed overlapping patient-level probabilities, partially entangled embeddings, and false-negative cases among participants with moderate-to-severe PHQ-8 scores, explaining why the model remained conservative at the fixed threshold. The multimodal fusion experiment further suggested that short text segments may introduce semantic noise when dialog-level context is unavailable.

Overall, the results provide preliminary evidence for the usefulness of multi-scale acoustic enhancement and emotion-reference contrastive calibration, but they do not support clinical diagnostic deployment. The main contribution of this work is therefore a structured short-segment acoustic screening framework with explicit patient-level uncertainty and error analysis, rather than a validated diagnostic system. Future work should improve depressed-case sensitivity, calibrate thresholds prospectively, stabilize training across random seeds, explore long-context acoustic–semantic modeling, and evaluate the framework on larger external and real-world cohorts before practical deployment. Detailed supplementary evaluation tables are provided in Appendix A.

## Figures and Tables

**Table 1:** DAIC-WOZ participant- and segment-level data distribution after preprocessing.

Split	Patients	Segments	Non-Dep. Patients	Dep. Patients	Overlap
Train	107	5128	77	30	0
Dev.	35	1917	23	12	0
Test	47	2680	33	14	0

**Table 2:** DAIC-WOZ symptom severity distribution at participant and segment levels.

Split	Normal Patients	Subclin. Patients	Mod./Sev. Patients	Normal Seg.	Subclin. Seg.	Mod./Sev. Seg.
Train	47	29	31	2318	1301	1509
Dev.	17	6	12	819	325	773
Test	22	11	14	1232	705	743

**Table 3:** MODMA participant- and segment-level data distribution after preprocessing.

Split	Patients	Seg.	HC Patients	MDD Patients	HC Seg.	MDD Seg.	Overlap
Train	35	1285	20	15	737	548	0
Dev.	6	239	3	3	118	121	0
Test	11	336	6	5	177	159	0

**Table 4:** MODMA symptom severity distribution at participant and segment levels.

Split	Non-Patients	Mild Patients	Mod. Patients	Severe Patients	Non-Seg.	Mild Seg.	Mod. Seg.	Severe Seg.
Train	18	2	2	13	691	46	97	451
Dev.	2	1	0	3	75	43	0	121
Test	6	1	1	3	177	34	11	114

**Table 5:** Performance comparison of depression recognition models.

Model Architecture	Accuracy	F1 Score	Non-Depressed Recall
Baseline (Wav2Vec)	0.5532	0.4324	0.55
Wav2Vec + MSC	0.6170	0.4375	0.67
Wav2Vec + MSC + ReCLR	0.7021	0.4167	0.85
Wav2Vec + MSC + ReCLR (MODMA)	0.7273	0.7273	0.74

**Table 6:** Experimental results of related methods.

Model	Accuracy	F1 Score	Non-Depressed Recall
Max + Logistic Regression	0.743	0.400	0.692
Max + Random Forest	0.657	0.415	0.656
Max + Gradient Boosting	0.657	0.143	0.460
Mean + Logistic Regression	0.638	0.308	0.483

**Table 7:** Methodological comparison with recent speech-based depression detection studies.

Study	Recent/Relevant Focus	Modality/Setting	Main Method	Comparison with Present Study
Huang et al. [10] (2024)	Pretrained speech representation for depression recognition	Speech; voice-based recognition	Voice pretraining model	Related acoustic pretraining evidence; different because it does not target 10 s participant-level screening.
Zhang et al. [9] (2024)	Low-resource acoustic depression detection	Speech; low-resource setting	Wav2Vec 2.0 transfer learning	Related SSL acoustic baseline; present study adds MSC and ReCLR calibration.
Han et al. [18] (2024)	Spatial–temporal speech modeling	Speech-based depression recognition	Spatial–Temporal Feature Network	Different temporal modeling strategy; present study is lighter and short-window-oriented.
Yue et al. [19] (2024)	Hierarchical temporal attention	Speech depression detection	Dynamic-window hierarchical transformer	Different hierarchy design; present study uses MSC plus BiLSTM-attention for short segments.
Wang et al. [20] (2025)	Crossmodal depression representation	Audio, video, and text; multimodal setting	Spatio-temporal ensemble and alignment	Different multimodal long-context setting; present study remains a preliminary acoustic screening.
Present study (2026)	Short-segment spontaneous-speech screening	DAIC-WOZ participant-independent testing; MODMA supplementary	Wav2Vec 2.0 + MSC + ReCLR	Preliminary specificity-oriented framework; not a validated diagnostic SOTA system.

**Table 8:** Patient-level classification metrics of the final MSC-ReCLR model on the DAIC-WOZ test set.

Acc.	Dep. Prec.	Dep. Sens.	Spec.	Dep. F1	Macro-F1	W-F1	AUC-ROC	AP
0.7021	0.5000	0.3571	0.8485	0.4167	0.6083	0.6858	0.5411	0.3926

**Table 9:** Patient-level confusion matrix of the final MSC-ReCLR model on the DAIC-WOZ test set.

Actual/Predicted	Non-Depressed	Depressed
Non-depressed	28	5
Depressed	9	5

**Table 10:** Patient-level bootstrap confidence intervals of the final MSC-ReCLR model on the DAIC-WOZ test set.

Metric	Point Estimate	95% CI Lower	95% CI Upper
Accuracy	0.7021	0.5745	0.8298
Sensitivity	0.3571	0.1000	0.6250
Specificity	0.8485	0.7097	0.9667
Depressed F1	0.4167	0.1250	0.6400
Macro-F1	0.6083	0.4503	0.7459
AUC-ROC	0.5411	0.3384	0.7333
Average Precision	0.3926	0.2070	0.6646

**Table 11:** Patient-level threshold sensitivity analysis of the final MSC-ReCLR model on the DAIC-WOZ test set.

Thresh.	Acc.	Sens.	Spec.	Dep. F1	TN/FP	FN/TP
0.30	0.5319	0.5000	0.5455	0.3889	18/15	7/7
0.35	0.5745	0.4286	0.6364	0.3750	21/12	8/6
0.40	0.6170	0.4286	0.6970	0.4000	23/10	8/6
0.45	0.6383	0.4286	0.7273	0.4138	24/9	8/6
0.50	0.7021	0.3571	0.8485	0.4167	28/5	9/5
0.55	0.6809	0.1429	0.9091	0.2105	30/3	12/2
0.60	0.6809	0.0714	0.9394	0.1176	31/2	13/1

**Table 12:** Comparison of recognition accuracy across different symptom severity groups.

Model Architecture	Normal Group	Subclinical	Moderate-to-Severe
Baseline (Wav2Vec)	0.3636	0.4545	**0.6429**
Wav2Vec + MSC	0.5000	0.6364	**0.6429**
Wav2Vec + MSC + ReCLR	**0.7727**	**1.0000**	0.3571

Note: Bolded values represent the optimal results for that metric. PHQ-8 scores of 0–4 represent the normal group, 5–9 are subclinical, and ≥10 are moderate-to-severe.

## Data Availability

The data used in this study were derived from publicly available resources. The DAIC-WOZ dataset is available at https://dcapswoz.ict.usc.edu/wwwdaicwoz/ (accessed on 30 November 2025), and the MODMA dataset is available at https://modma.lzu.edu.cn/data/application/ (accessed on 12 December 2025).

## Appendix A

**Table A1:** Detailed evaluation of the baseline model.

Metric	Precision	Recall	F1 Score	Support
Non-Depressed	0.78	0.55	0.64	33
Depressed	0.38	0.64	0.47	14
Accuracy			0.57	47
Macro Avg	0.58	0.59	0.56	47
Weighted Avg	0.66	0.57	0.59	47

**Table A2:** Ablation experiment results of MSC used alone.

Metric	Precision	Recall	F1 Score	Support
Non-Depressed	0.70	1.00	0.82	33
Depressed	0.00	0.00	0.00	14
Accuracy			0.70	47
Macro Avg	0.35	0.50	0.41	47
Weighted Avg	0.49	0.70	0.58	47

**Table A3:** Detailed evaluation of the final model.

Metric	Precision	Recall	F1 Score	Support
Non-Depressed	0.76	0.85	0.80	33
Depressed	0.50	0.36	0.42	14
Accuracy			0.70	47
Macro Avg	0.63	0.60	0.61	47
Weighted Avg	0.68	0.70	0.69	47

**Table A4:** Multimodal fusion experiment results.

Metric	Precision	Recall	F1 Score	Support
Non-Depressed	1.00	0.06	0.11	33
Depressed	0.31	1.00	0.47	14
Accuracy			0.34	47
Macro Avg	0.66	0.53	0.29	47
Weighted Avg	0.79	0.34	0.22	47

**Table A5:** Patient-level prediction examples.

Participant ID	Predicted Probability	PHQ-8 Binary	Predicted Label
2010003	0.7068	1	1
2010013	0.2351	1	0
2020011	0.3264	0	0
2030009	0.7233	0	1
